# Proper Indication of BRAF^V600E^ Mutation Testing in Fine-Needle Aspirates of Thyroid Nodules

**DOI:** 10.1371/journal.pone.0064505

**Published:** 2013-05-24

**Authors:** Jieun Koh, Jong Rak Choi, Kyung Hwa Han, Eun-Kyung Kim, Jung Hyun Yoon, Hee Jung Moon, Jin Young Kwak

**Affiliations:** 1 Department of Radiology, Research Institute of Radiological Science, Yonsei University, College of Medicine, Seoul, Korea; 2 Department of Laboratory Medicine, Yonsei University, College of Medicine, Seoul, Korea; 3 Biostatistics Collaboration Unit, Medical Research Center, Yonsei University, College of Medicine, Seoul, Korea; University Medical Centre Utrecht, The Netherlands

## Abstract

**Background:**

The aim of this study was to evaluate the proper indication of adjunctive BRAF^V600E^ mutation analysis at the time of ultrasound-guided fine-needle aspiration in the diagnosis of thyroid nodules.

**Methods:**

This study included 518 nodules in 479 patients who underwent ultrasound-guided fine-needle aspiration with BRAF^V600E^ mutation. We calculated and compared the diagnostic performances of cytology and cytology with BRAF^V600E^ mutation analysis to detect malignancy among thyroid nodules according to ultrasound features and size.

**Results:**

Sensitivity, negative predictive value, and accuracy of cytology with BRAF^V600E^ mutation analysis were significantly higher than those of cytology alone in thyroid nodules with suspicious ultrasound features, regardless of size. Diagnostic performances did not show significant differences between cytology and cytology with BRAF^V600E^ mutation analysis in nodules without any suspicious ultrasound features, regardless of size.

**Conclusion:**

The BRAF^V600E^ mutation analysis was a useful adjunctive diagnostic tool in the diagnosis of thyroid nodules with suspicious ultrasound features regardless of size.

## Introduction

Among the various molecular events related to thyroid cancer, BRAF^V600E^ mutation is a highly specific somatic mutation for papillary thyroid cancer [1]–[3]. An activating point mutation of the T1799A point BRAF gene results in a valine-to-glutamic acid replacement at amino acid V600, resulting in the constitutive tumorogenesis [1], [4]. The prevalence of the mutation in papillary thyroid cancer is highly variable especially according to region, ranging from 29% to 83% in different studies from different areas of the world [4]. Among patients diagnosed as papillary thyroid cancer in Korea, the prevalence of BRAF^V600E^ mutation has been reported up to 84% [4]–[8].

Although detecting BRAF^V600E^ mutation plays an additional role in the definitive diagnosis of thyroid nodules [8]–[10], performing routine BRAF^V600E^ mutation analysis in addition to fine-needle aspiration (FNA) may be questioned, when considering its cost-effectiveness. Therefore, a proper indication for performing additional BRAF^V600E^ mutation analysis to FNA is needed. Although a few studies demonstrated proper guidelines in selecting which thyroid nodules for testing BRAF^V600E^ mutation, these studies have mostly focused on ultrasound (US) features and the test point of analysis [4], [9], [11], [12]. When considering that the prevalence of BRAF^V600E^ mutation was higher in patients with papillary thyroid cancer >1 cm in size than in patients with papillary thyroid microcarcinoma [8], [13]–[15], size of the thyroid nodule can act as an indicative factor in deciding whether to perform additional BRAF^V600E^ mutation analysis to FNA. Therefore, this study was to investigate the proper indication of adjunctive BRAF^V600E^ mutation analysis at the time of ultrasound-guided fine-needle aspiration (US-FNA) in the diagnosis of thyroid nodules.

## Materials and Methods

The institutional review board of severance hospital approved of this retrospective observational study and required neither patient approval nor informed consent for our review of patients’ images and records. However, written informed consent was obtained from all patients for US-FNA and BRAF^V600E^ mutation analysis prior to each procedure as a daily practice.

### Study Population

**Figure 1:**
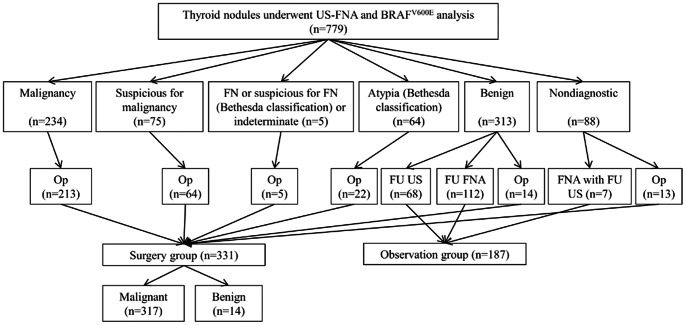
Diagram of study population. A total of 518 nodules in 479 patients were finally included in this study, and 331 nodules from 300 patients were confirmed pathologically, and 187 nodules from 182 patients were clinically observed by follow-up FNA (n = 112) or follow-up US (n = 75). Abbreviations: US-FNA, ultrasound-guided fine-needle aspiration; FN, follicular neoplasm; Op, operation; FU US, follow-up ultrasound; FU FNA, follow up fine-needle aspiration; FNA, fine-needle aspiration.

A total of 779 nodules in 722 patients who had US-FNA and BRAF^V600E^ analysis from January 2009 to October 2010 were initially enrolled in this study. Among them, 261 nodules were excluded for following reasons; further follow-up including second US or US-FNA was not performed (n = 191), follow-up US-FNA revealed cellular paucity, atypia, follicular or Hurthle cell neoplasm, suspicious malignancy, or malignancy but the patient had not undergone surgery (n = 66), follow-up US showed increase in size in nodules diagnosed as benign on cytology without further cytopathologic confirmation (n = 3), and missing radiologic reports (n = 1). A total of 518 nodules in 479 patients were finally included in this study (Figure 1). Of 518 nodules, 331 nodules from 300 patients were confirmed pathologically (Surgery group), and 187 nodules from 182 patients were clinically observed by follow-up FNA (n = 112) or follow-up US (n = 75). Mean period of follow-up US was 14.7 months. Two patients had two nodules each, of which one were pathologically confirmed after surgery and the other underwent observation. One patient had three nodules of which two were pathologically confirmed and the other underwent observation.

### US Analysis

For evaluation of the thyroid glands and cervical lymph nodes, a 5–12 MHz linear probe (iU22, Philips Medical Systems, Bothell, WA) or a 6–13 MHz linear probe (EUB-7500, Hitachi Medical, Tokyo, Japan) were used. Compound imaging was used in all images from the iU22 machine. Seven board-certified radiologists specialized in thyroid imaging with 1–15 years of experiences performed US and subsequent US-FNA.

US features of all thyroid nodules which had undergone US-FNA were prospectively recorded according to the internal component, echogenicity, margin, calcification, and shape at the time of US examination. Malignant US features were markedly hypoechogenicity, irregular or microlobulated margin, presence of microcalcifications, and taller than wide shape [16]. Thyroid nodules showing one or more suspicious US features described above were assessed as suspicious malignant, while those without any suspicious US features were assessed as probably benign [16]. Size of thyroid nodule was also recorded measuring the longest diameter.

### US-FNA and Cytologic Analyses

US-FNA was done by the same radiologist who performed US. Fine-needle aspiration was performed on the nodules showing suspicious features, and if none showed any suspicious US features, FNA was performed targeting at the nodule with the largest size. Each nodule was aspirated at least twice using freehand technique with a 23-gauge needle attached to 2-mL disposable plastic syringe. Obtained samples were expelled on to glass slides, smeared and placed immediately into 95% alcohol for Papanicolaou staining. One of the five cytopathologists specializing in thyroid cytology interpreted the smeared samples. Cytopathologists were not present during the biopsy procedures, but special staining was performed if needed. Cytology reports of June 2009 to November 2009 were based on the following 5 categories; 1) benign, 2) indeterminate cytology (follicular neoplasm or Hurthle cell neoplasm), 3) suspicious for papillary thyroid cancer, 4) malignant and 5) inadequate [9]. After December 2009 to the present, the Bethesda classifications are used in the cytology reports of thyroid aspiration studies [17].

BRAF^V600E^ mutation analysis was performed with the remaining material in the syringe used in aspiration. Remaining material was rinsed in 1 mL of normal saline, and was subjected to the BRAF^V600E^ mutation analysis.

### Dual Priming Oligonucleotide-based Multiplex Polymerase Chain Reaction (DPO-PCR)

BRAF^V600E^ mutation analysis using the DPO-PCR technology was performed using the Seeplex BRAFACE detection system (Seegene, Seoul, Korea) as described previously [13], [14].

### Data and Statistical Analysis

We used cytopathological results as the “gold standard”, pathologically confirmed malignancies classified into the positive group, and pathologically confirmed or clinical benign nodules classified into the negative group. Categorical data was summarized using frequencies and percentages. χ^2^ test was used in analysis of categorical variables, while independent two-sample *t* test was used in continuous variables.

The cytologic grouping and management was based on whether the recommendation was follow-up (clinical follow-up or repeat aspiration) or surgery, according to the Bethesda classifications [17]. Malignancy, suspicious for malignancy, indeterminate results from the study period before December 2009, suspicious for follicular neoplasm or follicular neoplasm on cytology results were included in the “positive” cytology group**.** The malignant cytologic diagnoses were considered positive cytology when calculating diagnostic values of FNA. To calculate diagnostic performances of FNA, we compared the results to the “gold standard”. True-positives (TP) were defined as nodules with “positive” cytology and a corresponding “positive” gold standard result. True-negatives (TN) had both “negative” cytology and gold standard one. False-negatives (FN) were defined as nodules with “negative” cytology and “positive” gold standard one. False-positives (FP) had positive cytology and “negative” gold standard one. To calculate diagnostic performances of FNA with BRAF^V600E^ mutation analysis, a nodule was considered “positive” group when either FNA or BRAF^V600E^ mutation was positive. The following statistical values were calculated as: Sensitivity = TP/(TP+FN) × 100; specificity = TN/(TN+FP) × 100; positive predictive value = TP/(TP+FP) × 100; negative predictive value = TN/(TN+FN) × 100; accuracy = (TP+TN)/(TP+TN+FP+FN) × 100. We calculated diagnostic performances of FNA and FNA with BRAF^V600E^ mutation analysis for detecting malignancy according to US features and size of the nodule. We also compared the diagnostic performances of FNA and FNA with BRAF^V600E^ mutation analysis for detecting malignancy according to US features and size, using logistic regression with generalized estimating equation method.

Analysis was performed using SAS software (version 9.1.3; SAS Institute, Cary, NC). Statistical significance was assumed when the *P* value was less than 0.05. All reported *P* values are 2-sided.

## Results

### Patient and Nodule Characteristics

Of the total 518 FNA with additional BRAF^V600E^ mutation analysis performed, 317 (61.2%) nodules were confirmed as malignancy, and the remaining 201 (38.8%) as benign (Table 1). The mean age of patients was 48.6±11.7 years. Size of the nodules ranged from 2 mm to 52 mm (mean 10.5±7.3 mm). Mean age of the malignant group (46.1±11 years) was younger than the benign group (52.7±11.6 years), showing statistical significance (*P*<0.001). Mean size of the benign nodules was 12.5±8.5 mm, which was larger than the malignant nodules (9.2±6.1 mm) with statistical significance (*P*<0.001).

**Table 1:** Cytological diagnoses of 518 nodules according to initial fine-needle aspiration results.*

Cytological diagnoses	Malignant			Benign		
	Total	BRAF^V600E^ mutation		Total	BRAF^V600E^ mutation	
		Positive	Negative		Positive	Negative
Non-diagnostic (n = 20)	13/20 (65)	1/13 (7.7)	12/13 (92.3)	7/20 (35)	0/7 (0)	7/7 (100)
Benign (n = 194)	10/194 (5.2)	4/10 (40)	6/10 (60)	184/194 (94.8)	2/184 (1.1)	182/184 (98.9)
Atypia (n = 22)	16/22 (72.7)	5/16 (31.3)	11/16 (68.8)	6/22 (27.3)	1/6 (16.7)	5/6 (83.3)
Follicular neoplasm or suspiciousfor follicular neoplasm (n = 5)	1/5 (20)	0/1 (0)	1/1 (100)	4/5 (80)	0/4 (0)	4/4 (100)
Suspicious for malignancy (n = 64)	64/64 (100)	27/64 (42.2)	37/64 (57.8)	0/64 (0)		
Malignant (n = 213)	213/213 (100)	139/213 (65.3)	74/213 (34.7)	0/213 (0)		
Total	317			201		

* Except where noted, data are number/total number (%).

Of the 317 nodules confirmed as malignant, 250 (78.9%) showed BRAF^V600E^ mutation. Among the 201 benign nodules, three (1.5%) showed BRAF^V600E^ mutation, two of which were pathologically confirmed as benign (adenomatous hyperplasia) by surgery, and one had undergone follow-up US for over a year with no interval change of size or characteristic.

### Analyses of All 518 Thyroid Nodules

Table 2 summarizes the diagnostic performances of FNA and FNA with BRAF^V600E^ mutation analysis. In all 518 thyroid nodules, additional BRAF^V600E^ mutation analysis improved sensitivity of FNA alone from 67.2% to 78.9% (*P*<0.001), accuracy from 79.9% to 86.5% (*P*<0.001), and negative predictive value from 65.9% to 74.7% (*P*<0.001). Specificity and positive predictive value did not show statistically significant differences between FNA and FNA with BRAF^V600E^ mutation analysis.

**Table 2:** Diagnostic performances of FNA and FNA with additional BRAF^V600E^ mutation analysis in the detection of malignancy according to US features and size of the nodules.* ^.^

		Sensitivity (%)	Specificity (%)	Accuracy (%)	PPV (%)	NPV (%)
**Overall (n = 518)**	**Total (n = 518)**					
	FNA	67.2 (213/317)	100 (201/201)	79.9 (414/518)	100 (213/213)	65.9 (201/305)
	FNA with BRAF^V600E^ mutation	78.9 (250/317)	98.5 (198/201)	86.5 (448/518)	98.8 (250/253)	74.7 (198/265)
	*P* value†	<0.001	0.081	<0.001	0.081	<0.001
	**Suspicious US feature (n = 386)**					
	FNA	68.7 (204/297)	100 (89/89)	75.9 (293/386)	100 (204/204)	48.9 (89/182)
	FNA with BRAF^V600E^ mutation	80.8 (240/297)	98.9 (88/89)	85 (328/386)	99.6 (240/241)	60.7 (88/145)
	*P* value	<0.001	0.315	<0.001	0.316	<0.001
	**Without suspicious US feature (n = 132)**					
	FNA	45 (9/20)	100 (112/112)	91.7 (121/132)	100 (9/9)	91.1 (112/123)
	FNA with BRAF^V600E^ mutation	50 (10/20)	98.2 (110/112)	90.9 (120/132)	83.3 (10/12)	91.7 (110/120)
	*P* value	0.306	0.154	0.563	0.121	0.422
**>10 mm (n = 175)**	**Total (n = 175)**					
	FNA	75.6 (62/82)	100 (93/93)	88.6 (155/175)	100 (62/62)	82.3 (93/113)
	FNA with BRAF^V600E^ mutation	84.1 (69/82)	97.8 (91/93)	91.4 (160/175)	97.2 (69/71)	87.5 (91/104)
	*P* value	0.006	0.153	<0.001	0.151	0.013
	**Suspicious US feature (n = 99)**					
	FNA	81.3 (61/75)	100 (24/24)	85.9 (85/99)	100 (61/61)	63.2 (24/38)
	FNA with BRAF^V600E^ mutation	89.3 (67/75)	100 (24/24)	91.9 (91/99)	100 (67/67)	75 (24/32)
	*P* value	0.012	–	0.013	–	0.016
	**Without suspicious US feature (n = 76)**					
	FNA	14.3 (1/7)	100 (69/69)	92.1 (70/76)	100 (1/1)	92 (69/75)
	FNA with BRAF^V600E^ mutation	28.6 (2/7)	97.1 (67/69)	90.8 (69/76)	50 (2/4)	93.1 (67/72)
	*P* value	0.295	0.151	0.563	0.046	0.405
**≤10 mm (n = 343)**	**Total (n = 343)**					
	FNA	64.3 (151/235)	100 (108/108)	75.5 (259/343)	100 (151/151)	56.3 (108/192)
	FNA with BRAF^V600E^ mutation	77 (181/235)	99.1 (107/108)	84 (288/343)	99.5 (181/182)	66.5 (107/161)
	*P* value	<0.001	0.315	0.094	0.316	<0.001
	**Suspicious US feature (n = 287)**					
	FNA	64.4 (143/222)	100 (65/65)	72.5 (208/287)	100 (143/143)	45.1 (65/144)
	FNA with BRAF^V600E^ mutation	77.9 (173/222)	98.5 (64/65)	82.6 (237/287)	99.4 (173/174)	56.6 (64/113)
	*P* value	<0.001	0.314	<0.001	0.316	<0.001
	**Without suspicious US feature (n = 56)**					
	FNA	61.5 (8/13)	100 (43/43)	91.1 (51/56)	100 (8/8)	89.6 (43/48)
	FNA with BRAF^V600E^ mutation	61.5 (8/13)	100 (43/43)	91.1 (51/56)	100 (8/8)	89.6 (43/48)
	*P* value	–	–	–	–	–

Abbreviations: FNA, fine-needle aspiration; US, ultrasound; PPV, positive predictive value; NPV, negative predictive value.

* Data presented in parentheses are number of nodules.

† *P* values were calculated using generalized estimating equation analysis.

Of the 386 nodules with suspicious US features, sensitivity, accuracy, and negative predictive value were higher in FNA with BRAF^V600E^ mutation analysis compared to FNA alone, 80.8% to 68.7% (*P*<0.001), 85% to 75.9% (*P*<0.001), and 60.7% to 48.9% (*P*<0.001), respectively, showing statistical significance. Specificity and positive predictive value did not show statistically significant differences between FNA and FNA with BRAF^V600E^ mutation analysis. In the 132 nodules without any suspicious US features, none of diagnostic performances showed statistically significant improvement with BRAF^V600E^ mutation analysis.

### Analyses of the 175 Nodules Larger than 10 mm

Sensitivity, negative predictive value, and accuracy of FNA with BRAF^V600E^ mutation analysis were higher than FNA alone (84.1% to 75.6%, 87.5% to 82.3%, and 91.4% to 88.6%) with statistical significance in the 175 thyroid nodules larger than 10 mm. Specificity and positive predictive value did not show statistically significant differences between FNA and FNA with BRAF^V600E^ mutation analysis.

Similar results were observed in the 99 nodules larger than 10 mm showing suspicious US features. Sensitivity, accuracy, and negative predictive value were improved in FNA with BRAF^V600E^ mutation analysis compared to FNA, 89.3% to 81.3% (*P = *0.012), 91.9% to 85.9% (*P = *0.013), and 75% to 63.2% (*P = *0.016), respectively. Specificity and positive predictive value show same values between FNA and FNA with BRAF^V600E^ mutation analysis. Of the 76 nodules larger than 10 mm without any suspicious US features, all values did not show statistically significant differences between FNA and FNA with BRAF^V600E^ mutation analysis.

### Analyses of the 343 Nodules Equal to or Smaller than 10 mm

Of the 343 nodules equal to or smaller than 10 mm, additional BRAF^V600E^ mutation analysis showed improvement to FNA in sensitivity (77% to 64.3%) and negative predictive value (66.5% to 56.3%), with statistical significance. Specificity, accuracy, and positive predictive value were not improved with additional BRAF^V600E^ mutation analysis, without statistical significance.

Diagnostic performances according to US features were analyzed among the 343 nodules. Among them, 287 were assessed as suspicious malignant, and the remaining 56 as probably benign. Sensitivity, accuracy, and negative predictive value were significantly improved in FNA with BRAF^V600E^ mutation analysis compared to FNA alone in the 287 nodules with suspicious US features, 77.9% to 64.4% (*P*<0.001), 82.6% to 72.5% (*P*<0.001), and 56.6% to 45.1% (*P*<0.001), respectively. Diagnostic performances showed similar values when comparing FNA to FNA with BRAF^V600E^ mutation analysis in the 56 thyroid nodules without any suspicious US features.

## Discussion

To the present date, FNA has shown acceptable diagnostic performances in the diagnosis of malignancy in thyroid nodules [18]–[20]. There are several limitations of FNA, however, such as false-negative and non-diagnostic results [20]. Many studies regarding molecular studies have been conducted to overcome these diagnostic limitations of FNA [11], [21], [22]. There have been several genetic abnormalities associated with thyroid carcinoma including point mutations such as those in the RAS and BRAF genes, and chromosomal rearrangements such as RET/PTC and PAX8/PPARγ [2]. Papillary carcinoma, the most common thyroid malignancy, harbors BRAF^V600E^, RET/PTC rearrangement, or the frequently found RAS mutations [23]. RAS genes and PAX8/PPARγ rearrangement are found more in follicular carcinomas [3], [23]. A recent study further demonstrated that BRAF^K601E^ was associated with the follicular variant type of papillary thyroid carcinoma [24]. Among them, BRAF^V600E^ mutation analysis showed good performances. However, when considering its cost-effectiveness, it is unclear whether if BRAF^V600E^ mutation analysis should routinely accompany US-FNA in the diagnosis of malignancy in patients with thyroid nodules. A proper indication for an adjunctive BRAF^V600E^ mutation analysis is required. Recent studies demonstrated that reflex molecular testing including the BRAF^V600E^ mutation can offer significant improvement in the preoperative diagnosis of thyroid cancer, especially in those showing indeterminate cytologic results including follicular lesion with atypia of uncertain significance, and suspicious for papillary carcinoma [25], [26]. Unfortunately, reflex molecular testing cannot always be adapted in all institutions, therefore, supporting the need for a proper guideline for the BRAF^V600E^ mutation analysis.

Several studies regarding proper indications for the additional BRAF^V600E^ mutation analysis demonstrated that this was more helpful when applied to nodules with suspicious features on US [4], [9], [11], [12], and at the time of initial US-FNA [9]. Also, the size of papillary thyroid cancer may affect the diagnostic performance of BRAF^V600E^ mutation analysis, when regarding the different prevalence of BRAF^V600E^ mutation in papillary thyroid microcarcinoma and papillary thyroid cancer larger than 10 mm [8], [13]–[15]. In this study, we investigated the diagnostic performance of FNA and FNA with BRAF^V600E^ mutation analysis to evaluate a proper indication for the BRAF^V600E^ mutation analysis, considering the size and US features of the thyroid nodules. Results of our study show that the prevalence of BRAF^V600E^ mutation was higher in papillary thyroid cancer (51/82, 62.2%) than papillary thyroid microcarcinoma (125/235, 53.2%). Sensitivity, accuracy, and negative predictive value of FNA with BRAF^V600E^ mutation analysis were significantly higher than those of FNA alone in thyroid nodules with suspicious US features, regardless of its size. All diagnostic performances of FNA with BRAF^V600E^ mutation analysis did not show significant differences compared to FNA alone in nodules without any suspicious US features, regardless of its size.

In the previous studies regarding the diagnostic performances of BRAF^V600E^ mutation analysis, specificity was reported to be almost 100% [12], but several false-positive cases were reported in Korea [7], [8], [10]. These false-positive cases reported in literature are thought to be caused by applying highly sensitive DPO-PCR or pyrosequencing analysis. These techniques focus on improving diagnostic sensitivity, which may result in false-positive cases [7]. In this study, three cases showed false-positive results among the 201 benign nodules; two of which were pathologically confirmed as benign by surgery, and one had undergone follow-up US for over one year. To reach 100% specificity to detect BRAF^V600E^ mutation at pyrosequencing, cut-off values were refitted to scarify sensitivity [7]. Further studies are required to evaluate the false positive results of BRAF^V600E^ mutation and consensus also should be needed to interpret and apply the results in patients with thyroid nodules.

There are several potential limitations to this study. First, the nodules which had not undergone surgery were included, based on the cytology results. This may have affected our results in ways of false-negative or false-positive cytologic results [27], [28]. Second, we divided groups based on the presence of suspicious US features. However, interobserver variability among radiologists in interpreting US images may have affected the results [29]–[31], which also may not be reproducible in other institutions. Third, sample size was different in thyroid nodules when grouped into those larger or equal to or smaller than 10 mm, which may have affected the results.

### Conclusion

The BRAF^V600E^ mutation analysis was a useful adjunctive diagnostic tool in the diagnosis of thyroid nodules with suspicious US features regardless of the size.
